# Evaluating Curvature-Induced Variation in Deep Learning-Based Beamforming for Flexible Transducers in Ultrasound-Guided Radiation Therapy

**DOI:** 10.3390/bioengineering13040398

**Published:** 2026-03-29

**Authors:** Ziwei Feng, Xinyue Huang, Hamed Hooshangnejad, Debarghya China, Junghoon Lee, Todd McNutt, Muyinatu A. Lediju Bell, Kai Ding

**Affiliations:** 1 Department of Radiation Oncology and Molecular Radiation Sciences, Johns Hopkins University School of Medicine, Baltimore, MD 21287, USA; zfeng15@jhmi.edu (Z.F.); hamed@jhu.edu (H.H.); junghoon@jhu.edu (J.L.); tmcnutt1@jhmi.edu (T.M.); 2 Department of Electrical and Computer Engineering, Johns Hopkins University, Baltimore, MD 21218, USA; 3 Department of Biomedical Engineering, Johns Hopkins University School of Medicine, Baltimore, MD 21287, USA; xinyue@gatech.edu (X.H.); dchina1@jhmi.edu (D.C.); 4 Department of Computer Science, Johns Hopkins University, Baltimore, MD 21218, USA

**Keywords:** ultrasound flexible transducer, machine learning, radiation oncology, ultrasound image reconstruction

## Abstract

Ultrasound imaging is a crucial tool for guiding radiation therapy, particularly for cancers such as pancreatic cancer, where tumors exhibit respiration-induced motion. While flexible ultrasound transducers offer improved anatomical conformity and reduced compression-induced distortion compared to rigid probes, their variable geometry presents significant challenges for conventional beamforming. In this study, we investigate a deep learning-based beamforming framework that directly predicts delayed RF data from raw RF input, bypassing explicit transducer shape estimation and traditional delay-and-sum computations. Building upon an artificial curvature simulation strategy, we systematically analyze the impact of curvature-induced variation and inherent RF noise on model performance and generalizability. We further introduce frequency-domain analysis to quantify RF-level signal variation that may not be apparent in spatial-domain image comparisons. Our results demonstrate that although noise-augmented training improves prediction consistency, reconstruction performance remains limited under the current prototype noise conditions. These findings highlight the importance of RF data diversity and noise characterization in developing clinically robust deep learning beamformers for flexible transducer-based ultrasound-guided radiation therapy.

## 1. Introduction

Pancreatic cancer is among the most prevalent and deadly cancer types [1] and ranks as the third leading cause of cancer-related deaths in the United States [2,3]. Stereotactic Body Radiation Therapy (SBRT) is a prominent form of treatment for this cancer [4,5]. It involves delivering a high dose of radiation to a small, well-defined tumor area with precise targeting, aiming to control tumor growth and improve patient survival rates. However, the effectiveness of SBRT in treating pancreatic cancers is often challenged by respiration-induced anatomical motion [2,6,7]. The proximity of lower-level abdominal organs and the minor anatomical shifts and deformations they undergo can displace the tumor from the targeted area and expose nearby radiosensitive organs at risk (OARs) to unintended radiation doses [2,6,8]. This displacement not only compromises tumor control but also increases the likelihood of OAR toxicity [9,10].

To mitigate these risks, typical clinical procedures involve setting a relatively safe prescription dose for treating the tumor or adjusting the planning target volume (PTV) coverage to protect OARs [11,12]. However, these measures can lead to insufficient tumor control. There are two primary solutions to safely administer a high treatment dose. The first involves the use of a physical spacer between the PTV and adjacent OARs, increasing the precision required in radiation delivery [13,14]. However, the applicability of spacers is limited by specific anatomical and safety considerations. The more widely applicable solution is image-guided radiation therapy, which allows for more accurate targeting and adjustment during treatment [15,16,17,18].

The integration of ultrasound guidance in radiotherapy is comparatively less widespread than X-ray-based image guidance systems, primarily due to limitations in transducer design. Traditional ultrasound transducers are highly operator-dependent, necessitating substantial training and additional personnel such as radiology technicians for clinical use [19,20,21]. Moreover, these transducers are typically encased in a rigid case, which, when pressed against the body, can cause anatomical alterations [19,21]. This variance in contact pressure and position during each radiotherapy session could lead to deviations in the delivered dose from the original treatment plan [22,23]. Consequently, there is a pressing need for innovative ultrasound transducer designs [20]. Such advancements are crucial for reducing operator dependency and minimizing anatomical changes, thereby improving the efficacy of image-guided monitoring of target motion during treatment [19,23].

In recent years, numerous research groups have introduced various innovative designs for ultrasound transducers, aimed at enhancing biomedical imaging and monitoring capabilities [24,25]. The development of a flexible array transducer represents a promising approach to target-tracking technology. The design of this flexible transducer overcomes the limitations of traditional rigid transducer arrays, specifically, issues related to operator dependency and anatomical deformation caused by compression when placing the transducer array on the patient’s surface [26,27,28]. Recently, a flexible array transducer designed as a patch was developed to replace mammography testing with a non-destructive method. Due to the wearable characteristics of the flexible transducer, which allow it to conform to the patient’s body shape, our group aims to apply this technology in radiotherapy for pancreatic and head and neck cancers [26]. However, this innovative flexible transducer introduces new challenges and stringent requirements for image reconstruction [29]. The need for a novel beamforming method arises due to its variable geometry in real time.

In recent years, significant progress has been made in deep learning-based ultrasound beamforming and reconstruction. Several studies have explored data-driven approaches to improve image quality, reduce computational burden, or compensate for hardware limitations [30,31,32,33]. In particular, recent work has investigated neural network-based delay estimation, adaptive beamforming, and geometry-aware reconstruction strategies for flexible or non-conventional transducer arrays [34,35,36,37,38]. Meanwhile, increasing attention has been paid to model robustness under realistic acquisition variability, including noise perturbations and signal-level variation [39,40]. Despite these advances, the impact of curvature-induced variation combined with inherent RF noise on direct RF-to-RF learning frameworks remains insufficiently explored.

Previously, we proposed an end-to-end deep learning approach to predict and re-construct ultrasound images and demonstrated its potential as a promising alternative to the conventional delay-and-sum (DAS) beamformer for the flexible transducer probe [41]. This method has shown promise in advancing the clinical application of flexible transducer technology in radiation therapy. To enhance the variety of training data, we introduced an artificial curvature method, which generates datasets with diverse “known” curvatures and their corresponding ground truth-delayed RF data used for reconstructing ultrasound images. However, this approach only increases the variety of transducer curvatures without introducing diversity in the raw radio-frequency (RF) signals, thereby limiting the model’s robustness. Specifically, in the previous framework, artificial curvature was applied to a limited set of original RF acquisitions. Although this process generated a larger number of paired datasets with varying geometric configurations, the underlying RF signal characteristics remained derived from the same small number of acquisition events. As a result, the generated samples were highly correlated at the signal level and lacked realistic acquisition-level variability, such as changes in system noise distribution or voltage-dependent signal variation.

In this study, we investigate how curvature-induced variation and inherent system noise in raw ultrasound RF data affect the performance and generalizability of deep learning-based beamformers for flexible transducer applications. Building upon our prior artificial curvature framework, we extend the analysis by focusing on the robustness and stability of deep neural networks trained under different noise conditions. The key contributions of this work are as follows: (1) we propose a direct RF-to-delayed-RF prediction strategy that bypasses traditional delay-and-sum computations and transducer shape estimation, thereby streamlining the image reconstruction process; (2) we evaluate model robustness by systematically injecting Gaussian noise into the training data and examining its effect on prediction fidelity and image quality; and (3) we introduce frequency-domain analysis as an additional evaluation metric to quantify the signal variation caused by both transducer curvature and system noise. Together, these contributions provide new insights into training deep learning-based beamformers under realistic data variation and advance the development of clinically viable ultrasound-guided radiation therapy using flexible transducers.

## 2. Materials and Methods

### 2.1. Experimental RF Data Acquisition

**Figure 1:**
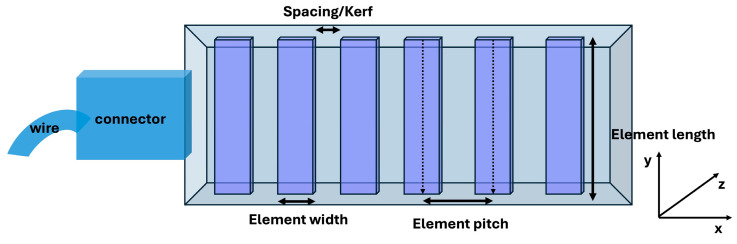
The geometric parameters of the flexible transducer.

In this study, we utilized a flexible transducer developed by Hitachi and Japan Probe (Tokyo, Japan). This transducer was connected to the Vantage 128 system (Verasonics Inc., Kirkland, WA, USA) to acquire RF data using a CIRS Small Part Ultrasound Phantom (Computerized Imaging Reference Systems Inc., Norfolk, VA, USA). The Vantage 128 system is equipped with 128 fully independent transmit and receive channels, a time-delay resolution of 4 ns, and 14-bit A/D converters sampling at 62.5 MHz. The system workstation included an Intel Xeon W-2155 CPU with 128 GB RAM (Intel Corporation, Santa Clara, CA, USA), running MATLAB (R2024b, MathWorks, Natick, MA, USA) for full control over sequence design and offline beamforming. Figure 1 and Table 1 display the geometric parameters of the flexible transducer.

For data collection, four experiments were conducted using two voltage settings (30 V and 50 V). (i) In the first experiment (Experiment 1), the transducer was placed on top of the phantom at a voltage setting of 30 V, and 24 RF datasets were acquired consecutively. (ii) The transducer was then removed and repositioned at the same location on the phantom, and an additional 24 RF datasets were acquired under the same voltage setting and nominally identical imaging conditions; this second acquisition was defined as Experiment 2. Thus, a total of 48 RF datasets were collected at 30 V. (iii) The same two-step procedure was repeated at 50 V: the first acquisition was defined as Experiment 3, and (iv) the repositioned acquisition was defined as Experiment 4, yielding another 48 RF datasets at 50 V. During each experiment, the transducer remained stationary on the phantom surface while the 24 RF datasets were acquired sequentially without intentional repositioning. Each RF dataset corresponds to a single acquisition frame under nominally identical imaging settings. Therefore, differences among the 24 datasets primarily reflect inherent system noise, electronic variability, and minor uncontrolled environmental perturbations (e.g., subtle mechanical vibrations), rather than deliberate changes in transducer geometry or imaging parameters. This acquisition design was intended to capture variability in RF data introduced by transducer repositioning and other acquisition-related factors under otherwise similar settings, providing a basis for evaluating signal-level variation relevant to developing a stable deep learning-based beamformer. All original RF acquisitions were performed with the flexible transducer in its flat (linear) configuration, corresponding to a linear array geometry prior to artificial curvature simulation.

### 2.2. Artificial Curvature Simulation and Dataset Generation

**Figure 2:**
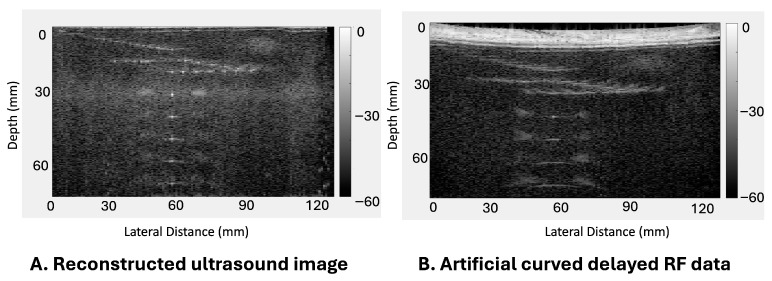
Example ultrasound images of (**A**) proper delayed ultrasound images of the phantom, (**B**) corresponding simulated RF data with convex transducer shape.

For each RF dataset, we generated 101 simulated before-delay RF datasets with convex transducer shapes, based on 96 different phantom scans of the same phantom, using a methodology we previously developed and tested [26]. This method computationally simulates the delay pattern corresponding to known transducer curvatures, allowing us to augment the data without physically deforming the transducer. Because the phantom has a rigid surface, it is not feasible to acquire actual curved transducer measurements on the same object. To briefly summarize this method, we employed a previously developed ‘simulated convex shape data’ method to increase the variety and quantity of training data by generating artificial RF data from the linearly collected RF data of the flexible transducer. The process involved (1) calculating the true Time of Flight (ToF) for each focal point based on the linear shape of the transducer, (2) calculating the curved ToF for each focal point based on a predetermined curve radius, and (3) delaying the original RF data from each channel accordingly. Figure 2 presents an example where Figure 2A illustrates the accurately delayed and reconstructed ultrasound images of the phantom, and Figure 2B shows the corresponding simulated delayed RF data with a convex transducer shape with a 600 mm radius. Consequently, we gathered a significant amount of paired training data, which includes simulated before-delay RF data with convex transducer shapes and their corresponding ground truth-delayed RF data for each focal point, or in other words, the simulated RF data with convex transducer shapes (pre-delay) and the ground truth of the properly delayed reconstructed ultrasound image (post-delay). Utilizing this method, we generated 101 sets of simulated RF data with different concave transducer curvatures ranging from 300 mm to 800 mm in 5 mm increments by scanning the phantom, serving as training data for this study. The selected curvature range (300–800 mm) represents both the mechanically feasible deformation limits of the flexible transducer and curvature scales relevant to abdominal surface geometry in tumor-tracking applications [26].

To organize the data for training, validation, and testing, we selected one RF dataset from each of the four experiments (Experiments 1, 2, 3, and 4 as described above) and generated the corresponding artificial curved RF data with the same curvature for validation. Similarly, another set of RF datasets from each experiment was used for testing. The remaining RF datasets were assigned to the training set. In total, 404 paired datasets were used for testing (101 from each experiment), and 404 paired datasets were used for validation. The remaining 8080 paired datasets were allocated to the training set. This corresponds approximately to an 80%, 10%, 10% split for training, validation, and testing, respectively.

### 2.3. Pipeline and Network

**Figure 3:**
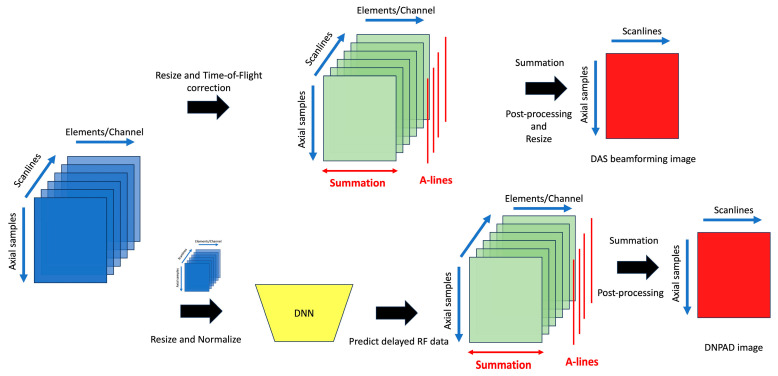
An overview of proposed processing pipeline. The DNN receives only resized and normalized RF data as input; no explicit curvature or transducer geometry information is provided.

Figure 3 depicts the comprehensive processing pipeline utilized in this study, which comprises two distinct paths. The first path serves as the benchmark for assessing the effectiveness of the proposed method and model. Here, the before-delayed RF channel data (pre-delay) are resized from a 3712 × 128 × 128 matrix to a 1024 × 128 × 128 matrix and delayed using the known ToF values to reconstruct the reference ultrasound image via conventional delay-and-sum (DAS) beamforming. This reconstruction employs conventional DAS beamforming techniques, including procedures for delay data and summation along the elements/channels. The second path introduces the proposed method via a deep neural network (DNN) model. Similarly, the before-delay RF channel data (pre-delay) are resized from a 3712 × 128 × 128 matrix to a 1024 × 128 × 128 matrix. Following normalization of all data to the range [0, 1], these data are input into the DNN, resulting in the generation of a predicted delayed RF matrix (post-delay) of size 1024 × 128 × 128. Finally, conventional summation is applied to the predicted delayed RF matrix to reconstruct the final B-mode ultrasound image named as the DNN image. It is important to note that the DNN is not provided with any explicit curvature or transducer shape information. All curvature-related effects are learned implicitly from the input RF data. We refer to images reconstructed from the DNN-predicted delayed RF matrix as DNPAD (Deep Neural Network–Predicted After-Delay), i.e., the network predicts the delayed RF data from pre-delay RF input, and conventional channel summation/DAS is then applied to form the B-mode image.

The Pix2Pix GAN architecture, incorporating a U-Net-based generator and a convolutional discriminator, is employed to estimate the delayed RF matrix from before-delay RF data. The training data consist of paired before-delay and ground truth-delayed after-delay RF datasets, with each scanline treated as an individual sample for input and output. This approach ensures high resolution and fidelity in the predicted RF matrices.

The generator utilizes a three-level encoder–decoder structure based on the U-Net framework. The encoder consists of multiple convolutional layers with 3 × 3 filters, batch normalization, and Rectified Linear Unit (ReLU) activation, followed by max-pooling layers with a 2 × 2 pooling and a stride of 2 to progressively reduce spatial dimensions. Starting with 1 channel, the encoder systematically increases the number of channels to 512 while reducing spatial dimensions from 128 × 1024 to 16 × 128. The bottleneck layer, located between the encoder and decoder, preserves data integrity through additional convolutional operations. The decoder mirrors the encoder but performs up-sampling using transposed convolutional layers, reducing the channel count while increasing spatial dimensions back to the original size of 128 × 1024. The final output layer applies a Sigmoid activation function to ensure normalized predictions for the delayed RF matrix.

The discriminator is a convolutional neural network designed to distinguish real from generated (fake) RF data. It comprises five convolutional layers with 4 × 4 kernels, each followed by batch normalization and LeakyReLU activation, except for the final layer, which employs a Sigmoid activation function to output a classification probability. This architecture provides robust feedback to guide the generator’s learning process, ensuring the generated outputs closely resemble the real data.

For testing the noise inherent in ultrasound RF signals, Gaussian noise is added to the before-delay RF data during training. Three variations of the Pix2Pix GAN are implemented. The baseline model excludes any noise addition, relying solely on clean before-delay RF data named as the Intrinsic Noise-Only (INO) model. In the second model, Gaussian noise with a standard deviation of 0.01 is applied, representing a relatively higher perturbation within the range tested (labeled the GN0.01 model). The third model introduces Gaussian noise with a lower standard deviation of 0.001 (the GN0.001 model) to evaluate the impact of minimal noise augmentation. These variations allow for a systematic assessment of the model’s noise-handling capabilities. Each acquisition stored only the first two frames; thus, temporal noise could not be estimated from repeated frames of the same scene. The Gaussian perturbations (σ = 0.001 and 0.01) were selected as bracketing stress-test levels to assess robustness under low- and moderate-noise conditions and are not intended to represent measured in vivo noise. The injected Gaussian noise experiments are included solely as controlled perturbations to illustrate robustness under additional uncertainty and are not intended to demonstrate overfitting behavior or measured temporal noise characteristics.

The loss function for the generator combines adversarial loss, binary cross-entropy loss (BCELoss), which encourages the generator to produce realistic outputs that can deceive the discriminator, and L1 loss, which minimizes pixel-wise differences between the predicted and ground truth delayed RF matrices. To prevent overfitting to noise, the weight of the L1 loss is reduced to 10. The discriminator uses BCELoss to measure its performance in distinguishing real from fake data. All models were implemented in PyTorch (v1.13.1) and trained on the Rockfish high-performance computing cluster using an NVIDIA A100 GPU with 40 or 80 GB memory (NVIDIA Corporation, Santa Clara, CA, USA). The initial learning rate was set to 1 × 10^−4^ with cosine annealing learning rate scheduling. The networks were trained for 100 or 200 epochs using the Adam optimizer (β_1_ = 0.5, β_2_ = 0.999) with a batch size of 4 to fit the memory constraints and ensure stable convergence.

### 2.4. Evaluation Metric

In this study, we used the 2D Discrete Fourier Transform computed via the Fast Fourier Transform (FFT), FFT shift, logarithmic scale transformation, and frequency difference computation to evaluate the signal and noise characteristics of the RF data in the frequency domain. The FFT is defined as

$$F\left(u,\nu \right)=\sum_{x=0}^{M-1}\sum_{y=0}^{N-1}f(x,y)\times e^{-j2\pi (\frac{ux}{M}+\frac{vy}{N})}$$

where *f*(*x*,*y*) represents the spatial-domain RF data, and *F*(*u*,*v*) represents the corresponding frequency-domain data.

The FFT shift is defined as

$$F\left(u,\nu \right)=\sum_{x=0}^{M-1}\sum_{y=0}^{N-1}f(x,y)\times e^{-j2\pi (\frac{ux}{M}+\frac{vy}{N})}$$

where *F*(*u*,*v*) represents the corresponding frequency-domain data after FFT.

The logarithmic scale transformation is defined as

$$Log-Scale\;FFT=log(1+\left|F_{shifted}(u,v)\right|)$$

The frequency difference computation is defined as

$$∆F\left(u,v\right)=\left|F_{shifted,1}\left(u,\;v\right)-F_{shifted,2}\;(u,v)\right|$$

Additionally, we used the Mean Squared Error (MSE) between ultrasound images reconstructed from ground truth-delayed RF data (using DAS) and those reconstructed from DNN-predicted delayed RF data to evaluate the accuracy of the model predictions.

$$MSE=\frac{1}{mn}\sum_{i=0}^{m-1}\sum_{j=0}^{n-1}{\left[I\left(i,j\right)-K(i,j)\right]}^{2}$$

where *I*(*i*,*j*) and *K*(*i*,*j*) are the pixel values of the DAS-reconstructed ultrasound image and the DNN-reconstructed image, respectively.

In this study, we consider that ultrasound RF data contain both inherent system noise and curvature-induced variation. While some signal differences result from the geometry-altering effect of artificial curvature simulation, others reflect the noise present in the original RF acquisition. Our evaluation does not attempt to isolate these sources explicitly but treats the observed variation as the combined effect of both. 

## 3. Results

**Figure 4:**
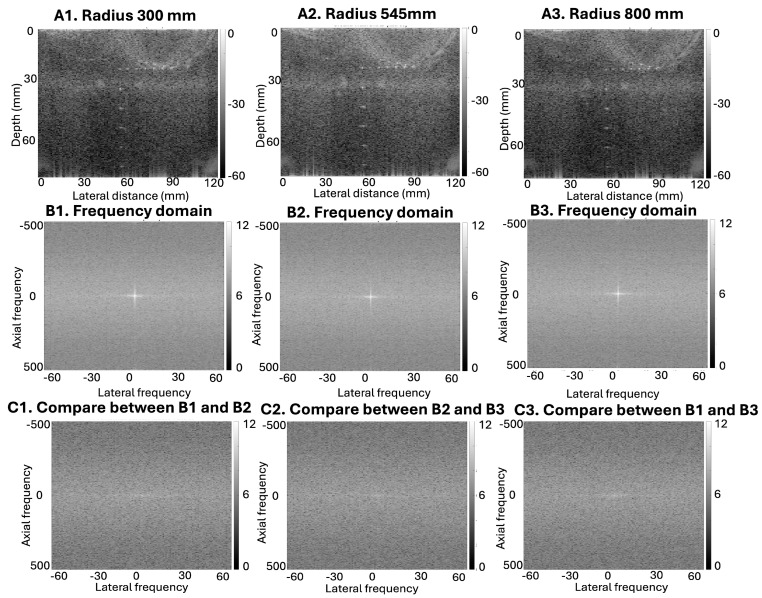
Frequency- and spatial-domain visualization of curvature-induced RF signal variation. Spatial-domain images (**A1**–**A3**) were reconstructed using conventional DAS beamforming with known artificial curvature parameters. The corresponding frequency-domain representations (**B1**–**B3**) were computed directly from the associated RF datasets prior to beamforming and DNPAD prediction. Subfigures (**C1**–**C3**) illustrate pairwise differences between the corresponding frequency-domain representations, specifically comparing B1 vs. B2 (**C1**), B2 vs. B3 (**C2**), and B1 vs. B3 (**C3**), to highlight curvature-induced variations.

As shown in Figure 4, direct visual differences across curvature conditions are limited in both the spatial-domain B-mode images and the corresponding Fourier-domain representations. However, subtraction-based and comparative frequency-domain analysis reveals curvature-dependent differences in RF signal content across these conditions.

Although B-mode images reconstructed using conventional DAS may not exhibit visually obvious differences due to envelope detection, log compression, and limited spatial resolution, the underlying RF signals contain subtle variations that are not readily appreciated by direct visual inspection in either representation but can be detected through comparative frequency-domain analysis of the RF data.

**Figure 5:**
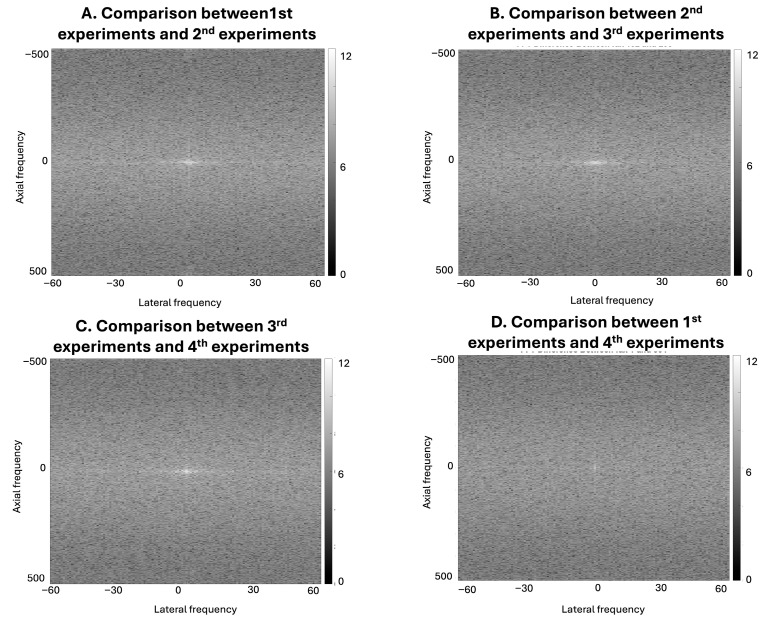
Frequency-domain visualization of RF signal variation across experimental configurations. Log-transformed absolute differences in the frequency domain were computed directly from raw RF datasets acquired under different experimental configurations but processed with the same artificial curvature. Panels (**A**–**D**) compare paired experiments: (**A**) Experiment 1 vs. Experiment 2 (both at 30 V), (**B**) Experiment 2 (30 V) vs. Experiment 3 (50 V), (**C**) Experiment 3 vs. Experiment 4 (both at 50 V), and (**D**) Experiment 1 (30 V) vs. Experiment 4 (50 V). The horizontal and vertical axes represent lateral and axial frequency components, respectively. These representations visualize RF-level variation across acquisitions and are not intended to assess spatial-domain image quality. All differences were computed directly from the raw RF datasets prior to any DAS reconstruction or DNPAD prediction.

The results in Figure 5 show RF-level signal variation across experimental acquisitions conducted under nominally similar conditions. Although the corresponding B-mode images from Experiments 1–4 appear visually similar in the spatial domain, the frequency-domain representations reveal differences in spectral content both within the same voltage setting and across different voltage levels. Direct visual differences are also limited in the corresponding Fourier-domain representations. In this study, RF-level variation was characterized through comparative frequency-domain analysis, including log-transformed difference maps, which demonstrated differences across acquisitions. These results show that acquisition-related factors, such as voltage setting and transducer repositioning, introduce measurable variation in the underlying RF signals. However, because the transducer was repositioned between voltage changes, the present experimental design does not allow complete isolation of voltage-specific effects from repositioning-related variability.

**Figure 6:**
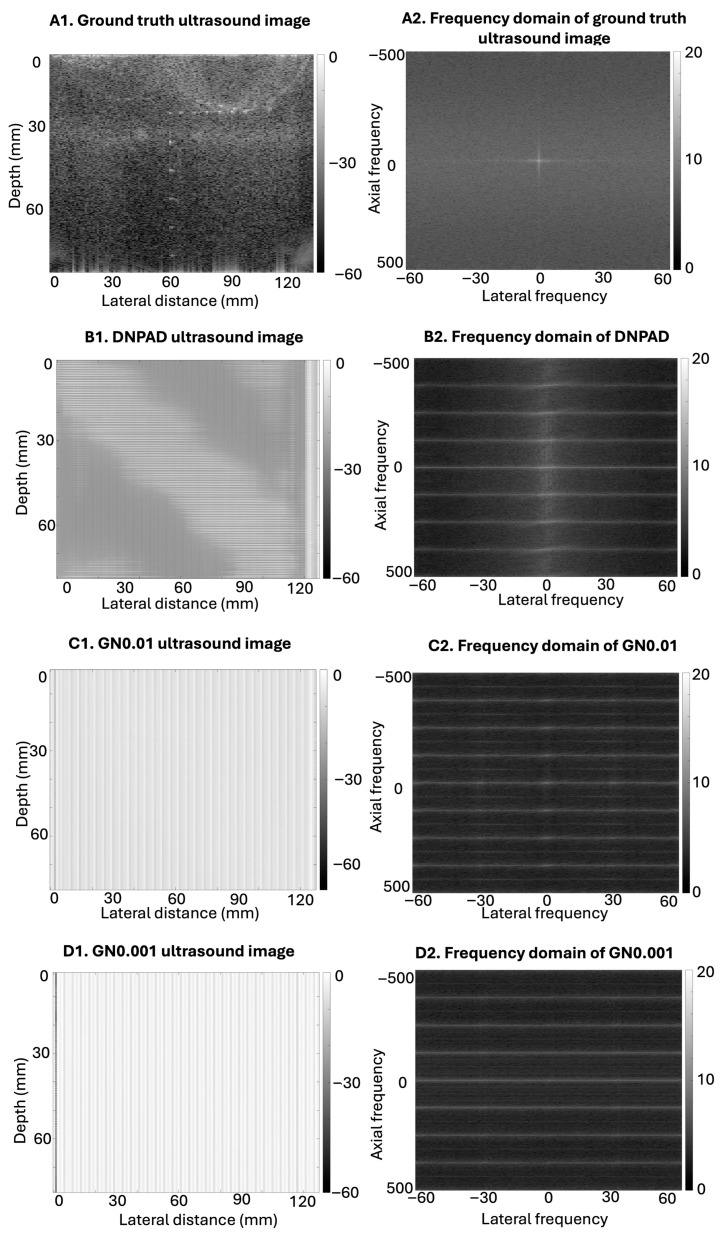
Spatial- and frequency-domain representations illustrating RF-level differences across DNPAD training strategies. The ground truth ultrasound image reconstructed from delayed RF data using conventional DAS beamforming (**A1**) and its corresponding frequency-domain representation (**A2**) are provided as references. Panels (**B1**,**C1**,**D1**) show spatial-domain images reconstructed from DNPAD-predicted delayed RF data using the INO, GN0.01, and GN0.001 training strategies, respectively. Panels (**B2**,**C2**,**D2**) display the corresponding frequency-domain representations computed from the predicted RF data.

Figure 6 provides a qualitative comparison of how different noise-augmentation strategies influence RF-level signal structure and the resulting reconstructed images. The spatial-domain images reconstructed from the INO, GN0.01, and GN0.001 models (B1, C1, D1) exhibit varying degrees of blurring and artifacts. However, these differences are subtle and difficult to interpret solely through visual inspection due to inherent noise and the limited size of the imaging target.

The corresponding frequency-domain analysis provides an additional signal-level comparison of the predicted RF data. Instead of relying solely on spatial-domain visualization, the frequency-domain representations illustrate differences in spectral organization and energy distribution across the three training conditions.

Accordingly, the frequency-domain analysis is presented to characterize RF-level signal variation across training conditions rather than to serve as a direct measure of spatial-domain image reconstruction quality.

**Figure 7:**
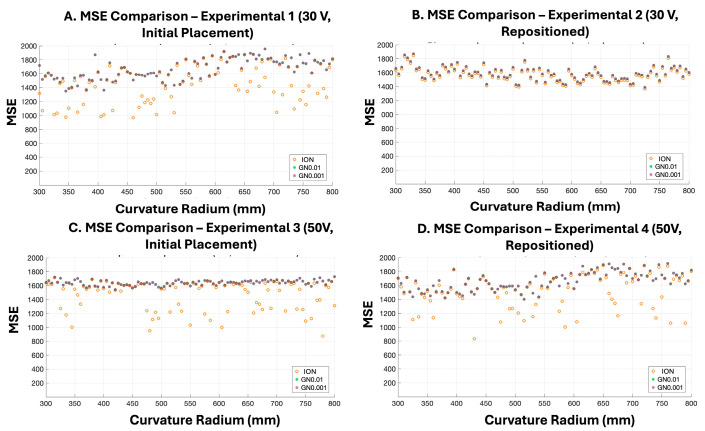
Comparison of Mean Squared Error (MSE) across testing datasets for different training strategies. MSE values were computed between ultrasound images reconstructed from DNPAD-predicted delayed RF data and corresponding ground truth images reconstructed from delayed RF data using conventional DAS beamforming. The 404 testing datasets were generated by applying artificial convex curvatures ranging from 300 mm to 800 mm (5 mm increments) to four original RF acquisitions obtained under different experimental conditions. Panels (**A**–**D**) correspond to (**A**) 30 V, initial placement; (**B**) 30 V, repositioned; (**C**) 50 V, initial placement; and (**D**) 50 V, repositioned. The *x*-axis represents curvature radius (mm), and the *y*-axis represents MSE. Orange markers denote INO results, green markers denote GN0.01 (σ = 0.01), and purple markers denote GN0.001 (σ = 0.001). Due to the close agreement between the GN0.01 and GN0.001 results, the corresponding green and purple markers may overlap in the plots, making the two distributions less distinguishable in certain regions.

Figure 7 compares MSE values across 404 testing datasets for the INO, GN0.01, and GN0.001 models. The INO model, trained without noise augmentation, exhibits greater variability in reconstruction error across curvature conditions, including both lower MSE values in certain cases and more pronounced outliers. In contrast, the noise-augmented models (GN0.01 and GN0.001) produce more tightly clustered MSE distributions across curvature conditions.

The GN0.01 and GN0.001 models show similar MSE distributions across the tested curvature conditions, indicating comparable reconstruction performance under the two noise-augmentation levels.

## 4. Discussion

This study has successfully demonstrated that the inherent curvature-induced variation within ultrasound RF data is a critical factor influencing the training of pixel-to-pixel DNN models for predicting delayed RF data and reconstructing ultrasound images using a flexible transducer. The periodic mosaic errors observed in the ultrasound images re-constructed from predicted delayed RF data are attributed to the inherent curvature-induced variation in the raw RF signals. These findings highlight the challenge of training an alternative beamformer DNN model, emphasizing the necessity of increasing the variety and diversity of RF data and the anatomical targets they contain. This is a pivotal step toward enabling the effective application of flexible transducers in radiation therapy, facilitating real-time monitoring of moving targets and improving the precision of delivered radiation doses.

Our proposed workflow involves developing a DNN-based beamformer that directly predicts the delayed RF matrix from raw input RF data, bypassing explicit estimation of transducer element positions or shapes, as shown in prior studies [42,43,44]. A key distinction of our method lies in its streamlined calculation process. While it does not introduce a new beamforming technique, our approach effectively bypasses the traditional delay computation required for predicting the transducer elements’ shape. Instead, the model is trained to directly predict the delayed ultrasound image matrix, eliminating the need for explicit delay matrix computation and external transducer shape tracking steps used in previous workflows. In our previous research, to track the shape of the flexible transducer in real time, we employed the NDI Passive Polaris Spectra optical tracking system (Northern Digital Inc., Waterloo, ON, Canada). The system utilizes dual infrared cameras and passive marker arrays to triangulate spatial positions with sub-millimeter accuracy (~0.5 mm). It operates at an update rate of 60 Hz and connects to the host workstation via USB through a Host USB Converter, enabling continuous monitoring of transducer curvature during acquisition. Although the proposed framework is not yet optimized for real-time video-rate implementation, it simplifies the reconstruction workflow by eliminating explicit delay matrix computation and external shape tracking steps. This improvement enhances the feasibility of applying our method in clinical settings. However, this approach presents a significant challenge: the need for a larger and more diverse set of raw RF training data. Although artificial curvature simulation can generate a large number of paired datasets, the underlying RF signal characteristics remain derived from a limited number of original acquisitions, resulting in highly correlated samples with limited intrinsic variability. The model must learn to extract meaningful signal information from the RF data amidst various curvature-induced variation, including not only the scanning material but also the inherent noise present in ultrasound RF data. The inconsistencies observed in some predicted ultrasound images reflect a fundamental challenge in ultrasound deep learning: small target regions introduce high variability in signal appearance, especially when affected by geometric distortion or noise. This sensitivity contrasts with other imaging modalities where targets may be larger or less affected by transducer positioning. Future work may benefit from more targeted augmentation strategies or domain-specific loss functions to improve stability in such settings.

Compared to our previous research [26,42], the RF data we collected is based on a phantom with smaller point targets. Since our flexible transducer is a prototype with a lower resolution for ultrasound imaging, it imposes higher accuracy requirements on the trained model while making significant and observable signals less pronounced. Under the same training architecture and datasets, results showed more prominent curvature-induced variation in this setup, likely due to lower signal intensity in the raw RF data. These variations are not solely due to geometric curvature; rather, our frequency-domain analyses also suggest that voltage settings can significantly modulate RF signal characteristics. As demonstrated in Figure 4, artificial curvature introduces RF-level signal variation even when spatial-domain B-mode images appear visually similar. This occurs because envelope detection and log compression suppress subtle RF variations during B-mode reconstruction, making such differences difficult to identify through direct spatial-domain inspection. Frequency-domain comparison of the RF signals, however, reveals curvature-dependent changes in spectral content that are not readily observable in the reconstructed B-mode images. These RF-level differences are particularly relevant for RF-to-RF learning frameworks such as DNPAD, where the models operate directly on RF signals rather than on envelope-detected images. Consequently, curvature-induced RF variability may influence the learning process even when spatial-domain image differences remain subtle. Figure 5 further shows that repeated acquisitions conducted under nominally similar experimental conditions can also introduce measurable RF-level variation. The frequency-domain comparisons reveal structured spectral differences both within the same voltage setting and across acquisitions performed at different voltage levels. Because the transducer was repositioned between voltage changes, these variations likely reflect a combination of acquisition-related factors rather than a single isolated source. Together, these observations indicate that both transducer geometry and acquisition variability contribute to differences in RF signal characteristics that may affect model training behavior.

In addition to comparing our current results with our previous work [26,42], it is worth noting that many other studies on flexible transducer beamforming adopt a different strategy. These methods first estimate the transducer element positions from RF data and then reconstruct ultrasound images based on the estimated positions [44,45]. Such approaches require ground truth element position data for evaluation, which is not produced in our framework. In contrast, our method bypasses the element position estimation step entirely and directly predicts the delayed RF data from raw input RF signals. Because of this fundamental difference in workflow, a direct experimental comparison with those methods is not feasible. Instead, our evaluation focuses on comparing the proposed method with the conventional DAS beamformer, demonstrating the clinical feasibility and robustness of the direct RF-to-delayed RF prediction strategy under realistic variations in data acquisition. We acknowledge that this study does not provide a cross-architecture comparison among multiple deep learning models, and this remains a limitation to be addressed in future work.

However, our study is not without limitations, which must be acknowledged and addressed in future research. One significant constraint is the limited scope of our training data. Utilizing only two voltage settings of simulated RF data with convex transducer shapes may not adequately represent the diversity of scenarios encountered in clinical ultrasound imaging. Variations in ultrasound echoes and the physical response characteristics of different subjects are critical factors that our current model may not sufficiently capture. To address this limitation, future studies should aim to incorporate a more ex-tensive and diverse dataset, including RF data obtained under varied operational settings, such as different Time Gain Compensation (TGC) configurations and a broader range of voltage settings. Expanding the dataset will not only enhance the model’s accuracy and robustness but also increase its applicability across a wider range of clinical scenarios.

Another limitation lies in the limited variety in simulated flexible transducer shapes. The study primarily focused on convex curvatures, which do not fully encompass the variety of transducer shapes encountered in clinical practice. Flexible transducers can adopt a wide range of shapes, making it challenging to capture or simulate RF data (both raw and delayed) that accurately reflects this diversity. While Gaussian noise was introduced during model training to simulate natural signal variability, we also observed additional variation in the RF and reconstructed data resulting from the artificial curvature process. This curvature-induced variation reflects both signal modulation due to geometry changes and artifacts from interpolation. Combined with inherent system noise in the original acquisition, these effects contribute to the spectral and spatial differences shown in our results, and we interpret them jointly rather than as isolated sources of error. The specific noise levels (σ = 0.01 and 0.001) were selected as bracketing values to probe model robustness rather than calibrated to actual in vivo conditions. The comparable performance observed between the GN0.01 and GN0.001 models suggests that noise augmentation may primarily contribute to stabilizing training dynamics rather than directly improving image fidelity under the current prototype noise conditions. Consistent with the observations in Figure 6, curvature-induced variation and injected noise produce distinct patterns in the RF signal representations, even when spatial-domain image differences remain subtle.

This behavior is also reflected in the quantitative evaluation shown in Figure 7. The MSE distributions across the testing datasets indicate that the noise-augmented models (GN0.01 and GN0.001) produce more stable reconstruction behavior compared with the INO model, which exhibits larger variability and occasional outliers across curvature conditions. The similar performance observed between the GN0.01 and GN0.001 models further suggests that moderate noise perturbation primarily contributes to stabilizing the training process rather than substantially altering reconstruction accuracy under the current experimental conditions. Because we did not record continuous RF sequences, temporal noise could not be directly measured, and the Gaussian σ values were not empirically tuned. Plus, because only the first two frames were stored per acquisition, temporal averaging over a large number of frames was not feasible, preventing direct estimation or suppression of temporal noise. Our flexible array is a prototype without vendor post-processing, so its raw RF noise floor may exceed that of commercial scanners. Future work will acquire short sequences to measure temporal noise, refine augmentation parameters, and expand the dataset to include a broader spectrum of flexible transducer shapes. Incorporating advanced tracking sensors to monitor transducer shapes in real time could further improve modeling and prediction accuracy [42,46]. Finally, a direct quantitative comparison of the RF noise floor between the prototype flexible array and standard rigid transducers was not available in this study and will be addressed in future work using repeated acquisitions and matched experimental settings.

## 5. Conclusions

This study systematically examined the impact of curvature-induced variation and inherent RF noise on deep learning-based beamforming for flexible transducer applications in ultrasound-guided radiation therapy. By implementing a direct RF-to-delayed-RF prediction framework, we eliminated the need for explicit transducer shape estimation and conventional delay matrix computation, thereby simplifying the reconstruction workflow for flexible array systems.

Although noise-augmented training strategies improved prediction consistency, reconstruction performance remained limited under the current prototype RF noise conditions. These findings highlight the challenges associated with training RF-to-RF beamforming networks under curvature-induced signal variation.

Future research should focus on several key directions. First, expanding the RF dataset to include more diverse acquisition conditions, voltage settings, and anatomical targets will be critical for improving generalizability. Second, direct measurement and modeling of temporal RF noise characteristics may allow more realistic augmentation strategies and improved robustness. Third, incorporating a broader range of flexible transducer geometries and acquisition scenarios will better reflect real clinical variability. Finally, optimizing computational efficiency and inference speed will be necessary to enable real-time implementation in clinical radiotherapy workflows.

Overall, the findings indicate that under the current prototype’s RF noise and curvature-induced variation conditions, the DNPAD framework does not yet achieve consistently robust reconstruction performance, highlighting important practical constraints and guiding future methodological refinement.

## Figures and Tables

**Table 1:** The parameters of the flexible transducer.

Parameters	Value
Element number	128
Transmit elements	1
Receive elements	128
Frequency	5 MHz
Element width	0.8 mm
Element pitch	1 mm
Element length	10 mm
Element Kerf	0.2 mm

## Data Availability

The data presented in this study are available on request from the corresponding author. The data are not publicly available due to the use of a prototype flexible transducer, which is not commercially available and subject to usage restrictions.
